# Latent toxoplasmosis, Cytomegalovirus, and Herpes Simplex Virus infections and risk of motorcycle accidents: A case-control study in a county with a high rate of motorcycle injuries in Iran

**DOI:** 10.1371/journal.pone.0307950

**Published:** 2024-08-22

**Authors:** Esmail Rayatdoost, Mahdi Chegin, Ali Taghipour, Enayat Shadmand, Fatemeh Rezaei, Shahab Falahi, Azra Kenarkoohi, Milad Badri, Kavous Solhjoo, Amir Abdoli

**Affiliations:** 1 Research Center for Noncommunicable Diseases, Jahrom University of Medical Sciences, Jahrom, Iran; 2 Department of Emergency Medicine, Jahrom University of Medical Sciences, Jahrom, Iran; 3 Zoonoses Research Center, Jahrom University of Medical Sciences, Jahrom, Iran; 4 Department of Parasitology and Mycology, Jahrom University of Medical Sciences, Jahrom, Iran; 5 Research Center for Social Determinants of Health, Jahrom University of Medical Sciences, Jahrom, Iran; 6 Zoonotic Diseases Research Center, Ilam University of Medical Sciences, Ilam, Iran; 7 Department of Microbiology, Ilam University of Medical Sciences, Ilam, Iran; 8 Medical Microbiology Research Center, Qazvin University of Medical Sciences, Qazvin, Iran; Shiraz University of Medical Sciences, ISLAMIC REPUBLIC OF IRAN

## Abstract

**Background:**

Road traffic injuries (RTIs) are among the most important issues worldwide. Several studies reported that infection with the neurotropic parasite *Toxoplasma gondii* (*T*. *gondii*) increased the risk of car accidents. In this study, our objective was to investigate the possible associations among latent *T*. *gondii*, Cytomegalovirus (CMV), and Herpes Simplex Virus (HSV) infections with the risk of motorcycle accidents in Jahrom (Fars Province), which is a county with a high rate of motorcycle accidents in Iran.

**Methods:**

In the setting of a case-control study; 176 motorcyclist men, including 88 survivors of motorcycle accidents and 88 motorcyclist without accidents, were considered as case and control groups, respectively. Rates of latent infections with *T*. *gondii*, CMV, and HSV were assessed by an enzyme-linked immunosorbent assay (ELISA).

**Results:**

Eleven of 88 (12.5%) in the case group and 22 of 88 (25.0%) in controls were positive for anti-*T*. *gondii* IgG antibodies, this difference was statistically significant (OR = 0.42; CI: 0.19–0.95, *p* = 0.03). The general seroprevalence of CMV (94.3% in the case group *vs*. 87.5% in the control group, OR = 2.37; CI: 0.78–7.13, *p =* 0.12) and HSV (63.6% in the case group *vs*. 62.5% in the control group, OR = 1.05; CI: 0.57–1.94, *p* = 0.87) were not significantly different between the case and control groups.

**Conclusions:**

Although latent toxoplasmosis has been associated with traffic accidents in recent reports, we found a negative association between latent toxoplasmosis and motorcycle accidents among survivors of these accidents. As such, latent CMV and HSV infections did not differ significantly between the cases compared to the control groups.

## Introduction

Road traffic injuries (RTIs) are a worldwide public health issue with and are among the five primary causes of deaths annually. The rate of RTIs is projected to increase until 2030 and become one of the five causes of death in the world [1]. In Iran, the second leading cause of death is RTIs [2], which predominantly relates to motorcyclist [3]. Although the number of motorcycles is less than that of cars in Iran, most instances of morbidity and mortality are reported for motorcyclists, particularly among men aged 15 to 29 years and in urban areas [3–5]. The World Health Organization (WHO) classified motorcycle riders as a high-risk injury group, with approximately 49% of all trauma victims worldwide annually [6]. The risk of injuries due to motorcycle accidents is about ten times higher than car accidents [7].

Alcohol and substance abuse (ASA) is the main risk factor of RTIs among drivers [8–10]. Recent reports revealed that a decrease in driver cognitive capacity increases susceptibility to driving errors [11–13]. On the other hand, the association between latent neurotropic infections, such as infection with the protozoan parasite *Toxoplasma gondii* (*T*. *gondii*) [14–18], as well as infection with the viral pathogens [19, 20] such as Cytomegalovirus (CMV) [19–21] and Herpes Simplex Virus (HSV) [22] with several psychiatric disorders (i.e., anxiety and depressive disorders, obsessive-compulsive disorder (OCD)) and cognitive declines) have been documented. Recent studies also demonstrated a significant correlation between latent toxoplasmosis and the risk of traffic accidents [23–29]. However, there are no reports on the association between traffic accidents and CMV and HSV infections. As these infections amplify inflammatory immune responses, and given that inflammatory chemokines and cytokines play roles in various neurobiological pathways, it is plausible to consider a link between *T*. *gondii*, CMV, and HSV infections and the risk of RTIs. Considering the fact thatmotorcycle accidents are among the serious problems in Iran, our objective was to investigate the correlation between *T*. *gondii*, CMV, and HSV infections and motorcycle accidents in Jahrom (Fars Province), a county with a high rate of motorcycle injuries in Iran.

## Methods

### Ethical aspects

First, the study procedures and purpose were explained to all participants or parent(s), if the participant was a minor, and then written informed consent was obtained from them. The Research and Ethics Committee of Jahrom University of Medical Sciences approved the study protocols and procedures (ethics code: IR.JUMS.REC.1399.138).

### Study protocol

Strengthening the reporting of observational studies in epidemiology (STROBE) statement: guidelines for reporting observational studies for each section of the study (S1 Checklist) [30]. All data are fully available without restriction on FigShare (https://doi.org/10.6084/m9.figshare.25003058).

### Study population and samples

This is a case-control study that was carried out in Jahrom county (Fars province, south of Iran, coordinates: 28°38′N 53°22′E). Our data covers a period of one year from January 01, 2022 to December 31, 2022, which resulted in a sample of 88 men with a history of road accident. In addition, our control group was composed of individuals referred to the same hospital for routine laboratory tests. These control cases were motorcyclists, but without a road accident. The control group was matched with our case group for sex and age. Therefore, the case group included 88 motorcyclist men with nonfatal accidents (mean age 29.86 ± 30.43 years). The drivers were sent to the emergency department of the Peymaniyeh hospital (in Jahrom) for medical attention. The control group consisted of 88 motorcyclist (mean age 28.12 ± 28.53 years). In addition, a questionnaire was used for collecting the necessary information from these participants.

### Serological test for *T*. *gondii*, CMV, and HSV antibodies

The IgG antibodies of *T*. *gondii*, CMV and HSV were determined using the commercial enzyme-linked immunosorbent assay kits (ELISA) (Pishtaz Teb, Tehran, Iran), according to the manufacturer’s protocol. The HSV kit detected antibodies against both HSV-1 and HSV-2 viruses. Diagnostic criteria for IgG antibodies were determined by cut-off values; in all three infections, a result of 11 IU / ml or more was considered positive, while a result below 11 IU / ml was considered negative (Pishtaz Teb, Tehran, Iran).

### Statistical analysis

In this study, the case and control groups were divided into four age groups: 14–25 years, 26–35 years, 36–45 years and 46–56 years, and the number and percentage of infected subjects were identified. Logistic regression was used to calculate the odds ratios (OR) and confidence intervals (CI) for the aforementioned three types of infection, according to the age groups. Data analysis was performed with SPSS version 23 software. *P* values lower than 0.05 were considered statistically significant in this study.

## Results

### General characteristics of the participants

A total of 176 male participants, including 88 motorcyclists who had a road accident and 88 control riders, were included in this study. The mean age was 29.86 ± 30.43 years in motorcyclist involved in accidents *vs*. 28.12 ± 28.53 years in control motorcyclist (S1 Table). Most of the participants were in the age range of 14–25 (52 individuals in the case group *vs*. 53 in the control group) and the smallest number of participants were in the age range of 46–56 (5 individuals in the case group *vs*. 4 in the control group).

### Frequency of anti-*T*. *gondii* IgG antibodies

**Fig 1:**
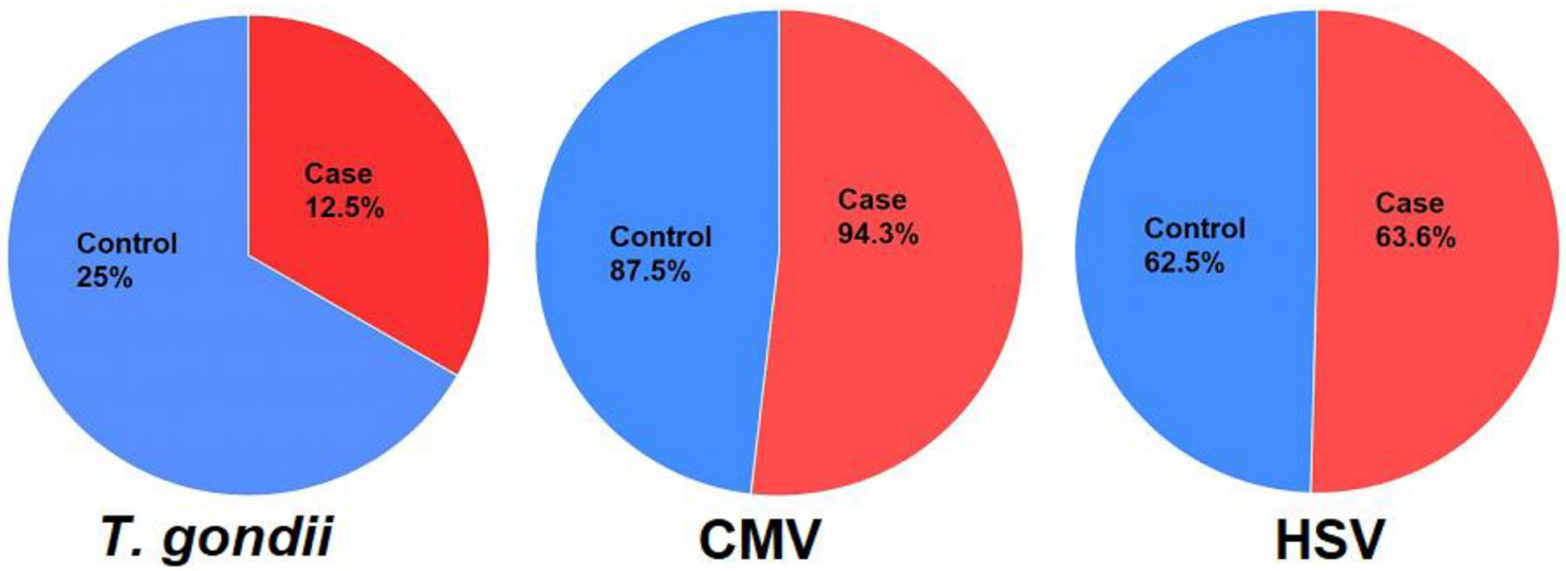
Seroprevalence (percentages) of *T*. *gondii* (*p* = 0.03), CMV (*p* = 0.12), and HSV (*p* = 0.87) in case and control group.

**Fig 2:**
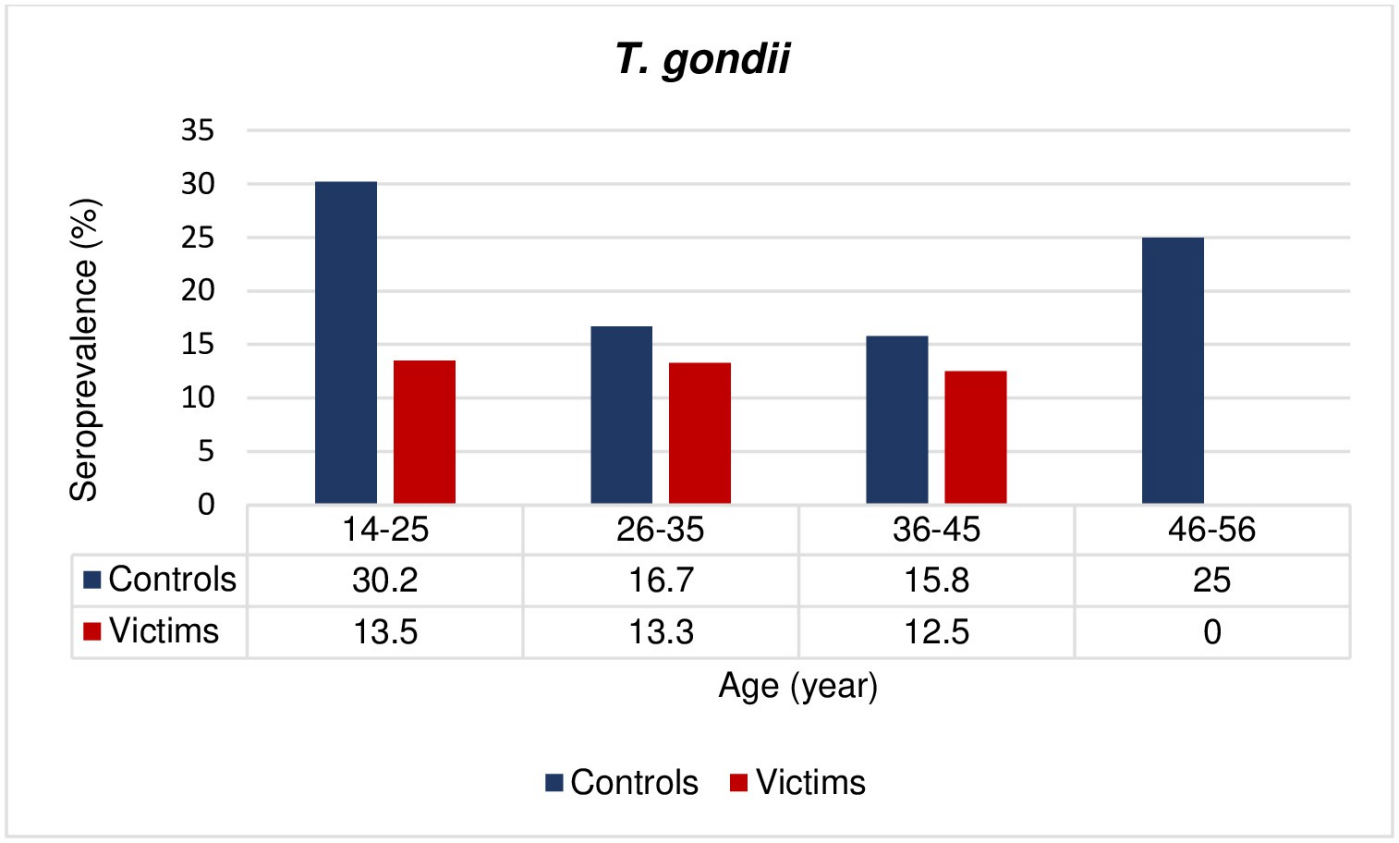
Seroprevalence (percentages) of *T*. *gondii* infection according to age group. P value was statistically significant in the age group of 14–25 (*p* = 0.04).

Eleven out of 88 (12.5%) in the case group and 22 out of 88 (25.0%) in controls were positive for anti-*T*. *gondii* IgG antibodies, this difference was statistically significant (OR = 0.42; CI: 0.19–0.95, *p* = 0.03) (Table 1 and Fig 1). The majority of seropositive participants were in the age range of 14–25, representing 13.5% and 30.2%, in the case and control groups, respectively (Table 1 and Fig 2).

**Table 1:** Seroprevalence of *T*. *gondii*, CMV, and HSV based on age groups.

Infections	Age groups (year)	Case group, N (%)	Control group N (%)	Odds Ratio	C.I. (95%)	*p*
***T*. *gondii***	14–25	52 (7, 13.5)	53 (16, 30.2)	0.36	0.13–0.96	**0.04**
	26–35	15 (2, 13.3)	12 (2, 16.7)	0.77	0.09–6.44	0.81
	36–45	16 (2, 12.5)	19 (3, 15.8)	0.76	0.11–5.23	0.78
	46–56	5 (0, 0%)	4 (1, 25)	0.21	0.006–6.81	0.38
	Total prevalence	88 (11, 12.5)	88 (22, 25.00)	0.42	0.19–0.95	**0.03**
**CMV**	14–25	52 (48, 92.3)	53 (47, 88.7)	1.53	0.41–5.77	0.53
	26–35	15 (14, 93.3)	12 (11, 91.7)	1.27	0.07–22.72	0.86
	36–45	16 (16, 100.0)	19 (15, 78.9)	9.58	0.47–192.99	0.14
	46–56	5 (5, 100.0)	4 (4, 100.0)	1.22	0.02–74.73	0.92
	Total prevalence	88 (83, 94.3)	88 (77, 87.5)	2.37	0.78–7.13	0.12
**HSV**	14–25	52 (33, 63.5)	53 (32, 60.4)	1.13	0.52–2.50	0.74
	26–35	15 (8, 53.3)	12 (10, 83.3)	0.22	0.04–1.1	0.11
	36–45	16 (11, 68.8)	19 (11, 57.9)	1.6	0.40–6.45	0.51
	46–56	5 (4, 80.0)	4 (2, 50.0)	4.0	0.21–75.66	0.35
	Total prevalence	88 (56, 63.6)	88 (55, 62.5)	1.05	0.57–1.94	0.87

### Frequency of anti-CMV IgG antibodies

**Fig 3:**
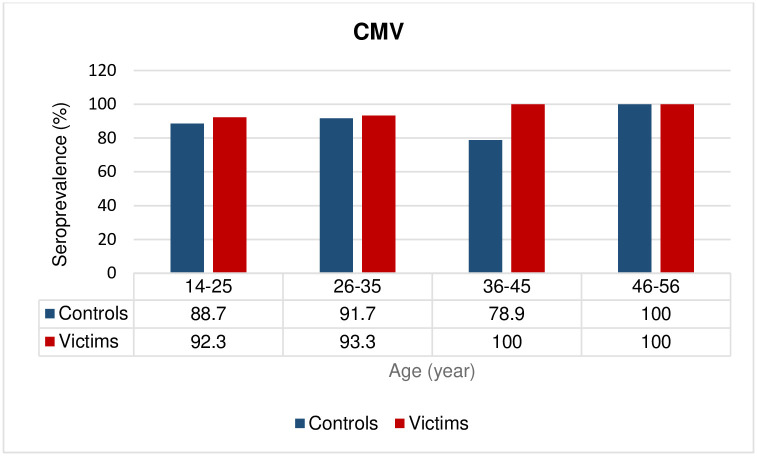
Seroprevalence (percentages) of CMV infection according to age group. *P* values were not statistically significant in all age groups.

The frequency of anti-CMV IgG antibodies in the case group (94.3%) was higher than in the control group (87.5%), although no statistically significant differences were found (OR = 2.37; CI: 0.78–7.13, *p =* 0.12) (Table 1 and Fig 1). There were no significant differences in the frequencies of anti-CMV IgG antibodies between different age strata (Table 1 and Fig 3).

### Frequency of anti-HSV IgG antibodies

**Fig 4:**
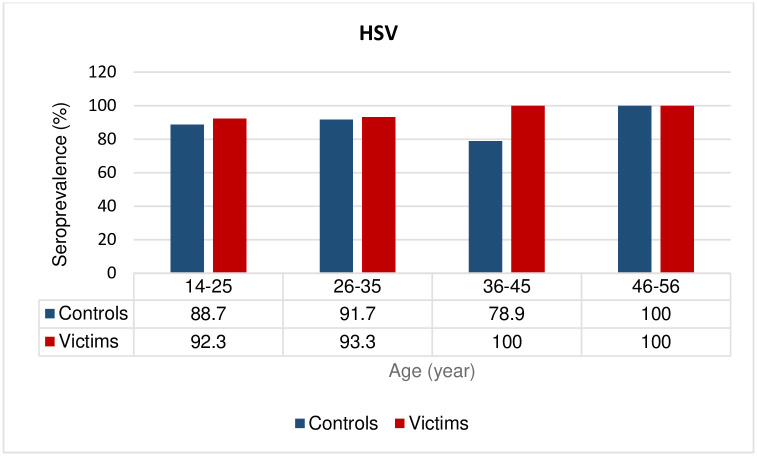
Seroprevalence (percentages) of HSV infection according to age group. *P* values were not statistically significant in all age groups.

Fifty-six out of the 88 (63.6%) individuals in the case group and 55 of the 88 (62.5%) in the control group were positive for anti-HSV IgG antibodies; however, this difference was not statistically significant (OR = 1.05; CI: 0.57–1.94, *p* = 0.87) (Table 1 and Fig 1). There were no significant differences in the frequencies of anti-HSV IgG antibodies between different age strata (Table 1 and Fig 4).

## Discussion

The current study shows that anti-*T*. *gondii* IgG antibodies were significantly lower in the case than in the control groups. The first report linking *T*. *gondii* infection with traffic accidents was presented by Flegr et al. [27] in the Czech Republic. [24]. They reported that subjects with latent toxoplasmosis had a 2.65 times higher risk of an accident compared to subjects without latent toxoplasmosis. Another investigation by the same group of researchers [25], who performed a prospective cohort study on traffic accidents and latent toxoplasmosis, demonstrated a higher risk of traffic accidents in subjects infected with *T*. *gondii* infected with *T*.*gondii* than in individuals without toxoplasmosis [25]. Two studies in Turkey revealed a significantly higher risk of car accidents among individuals with latent toxoplasmosis compared to the *T*. *gondii-*negative control group [27, 29]. Galván-Ramírez et al. [26] in Mexico reported that car accident drivers with high anti-*T*. *gondii* antibody titers had a significantly higher rate of traffic accidents compared to low anti-*T*. *gondii* antibody titers. Another study in the Russian Federation revealed that car drivers with latent toxoplasmosis had a 2.37 times higher risk of road accidents compared to the *T*. *gondii-*negative control group [28]. However, unlike the studies reported, our results showed a lower prevalence of latent toxoplasmosis in the case group when compared to the control group. The total rate of seroprevalence of toxoplasmosis among the general population and Iranian blood donors is 39.3% (95% CI = 33.0%–45.7%) [31] and 32.9% (95% CI: 25.3%–41.6%) [32], respectively. The Jahrom County has a hot semi-arid climate and average rainfall about 285 millimeters (11.2 in) per year (https://en.wikipedia.org/wiki/Jahrom). This condition could justify the lower prevalence rate of toxoplasmosis in Jahrom County than the overall prevalence in Iran. It is possible that the prevalence of toxoplasmosis is lower among motorcycle accident survivors because *T*. *gondii*-infected individuals are less likely to desire or have the courage to obtain a motorcycle license, probably due to the observed lower levels of novelty seeking trait in those infected with this parasite [16, 33]. Another possibility is that motorcyclists infected with *T*. *gondii* tend to have more severe traffic accidents compared to uninfected motorcyclists, often resulting in death or such severe health consequences that they were unable to provide informed consent to participate in the study. Such risky driving behavior could be related to their lower willingness to adhere to social norms (including traffic regulations) [34–36] or to increased testosterone levels in infected individuals [37–39] and male rats [40].

We hypothesized that other latent neurotropic infections, CMV and HSV, could be involved as a risk factor for accident. However, the results did not show a significant difference between the case and control groups in terms of the CMV and HSV IgG antibodies.

Infections may have a dual role in the etiology of noncommunicable diseases. On the one hand, infection with *T*. *gondii*, CMV, and HSV has been reported to be associated with an increased risk of some neuropsychiatric disorders [19, 20, 41–45]. On the other hand, in some studies, it is reported that infections with specific latent pathogens, such as *T*. *gondii*, CMV and HSV, can ameliorate the immune response away from inflammatory conditions; this concept is known as the ’hygiene hypothesis’. For example, latent CMV and HSV infections have been reported to play a protective role against atopy and allergic asthma [46–48], and coronary heart disease [49]. Latent toxoplasmosis has been reported to play a protective role against Multiple Sclerosis (MS) [50–52] and in mouse models of Alzheimer’s disease [53, 54]. Hence, it is plausible to hypothesize that the observed higher prevalence of *T*. *gondii* in the control group compared to traffic accident participants can be explained within the framework of the hygiene hypothesis.

### Strength, limitations, and future directions

This study has some strengths and limitations. To our knowledge, this is the first study to evaluate the rate of latent infections among individuals with motorcycle accidents. As such, this is the first study to assess the rate of latent CMV and HSV infections in the field of traffic accidents. The major limitation of this study is its small sample size and the lack of sampling from individuals who died following accident. Therefore, we suggest that more studies with a larger sample size should be conducted to increase our knowledge about the role of latent infections in traffic accidents.

## Conclusions

This study reveals that latent infection with *T*. *gondii* is associated with a lower risk of accident among motorcycle riders, while CMV and HSV infections did not differ significantly between the cases and control groups. Since this is the first study to assess the association between latent toxoplasmosis and accident rates among motorcyclists, our results should be interpreted with caution; therefore, we recommend more studies in this regard.

## Supporting information

S1 Checklist STROBE statement—Checklist of items that should be included in reports of case-control studies. (DOC)

S1 Table Age groups of case and control groups. (DOCX)

## Abbreviations

**ASA**: alcohol and substance abuse
**Aβ**: amyloid β
**CI**: confidence interval
**CMV**: Cytomegalovirus
**ELISA**: enzyme-linked immunosorbent assay
**HSV**: Herpes Simplex Virus
**MS**: Multiple Sclerosis
**OCD**: obsessive-compulsive disorder
**RTIs**: Road traffic injuries
**STROBE**: Strengthening the Reporting of observational studies in epidemiology
*T. gondii*: *Toxoplasma gondii*
**WHO**: World Health Organization
