# Supplementary material for: Latent toxoplasmosis, Cytomegalovirus, and Herpes Simplex Virus infections and risk of motorcycle accidents: A case-control study in a county with a high rate of motorcycle injuries in Iran
Source: PLoS One. 2024 Aug 22;19(8):e0307950. doi: 10.1371/journal.pone.0307950 (PMC11341033; doi:10.1371/journal.pone.0307950)
Supplement: S1 Table — (DOCX) [file pone.0307950.s002.docx]

**Supplementary Table 2.** Age groups of case and control groups.

| **Age groups (year)** | **Case group, N (%)** | **Control group N (%)** |
| --- | --- | --- |
| 14-25 | 52 (59.09 %) | 53 (60.22%) |
| 26-35 | 15 (17.04%) | 12 (13.63%) |
| 36-45 | 16 (18.18%) | 19 (21.59%) |
| 46-56 | 5 (5.68%) | 4 (4.54%) |
| **Mean age** | 29.86 ± 30.43 | 28.12 ± 28.53 |
| **Total** | 88 | 88 |
